# Draft Genome Sequence of the Thermophilic Acetogen *Moorella humiferrea* DSM 23265

**DOI:** 10.1128/genomeA.00357-18

**Published:** 2018-04-26

**Authors:** Anja Poehlein, Alisa Keyl, Jan C. Milsch, Rolf Daniel

**Affiliations:** a Genomic and Applied Microbiology & Göttingen Genomics Laboratory, Institute of Microbiology and Genetics, Georg-August University of Göttingen, Göttingen, Germany; b Members of the Applied Bioinformatics in Microbiology Course of the Molecular Life Sciences: Microbiology, Biotechnology and Biochemistry M.Sc./Ph.D. program, Georg-August University of Göttingen, Göttingen, Germany

## Abstract

Moorella humiferrea is an endospore-forming, anaerobic, and thermophilic bacterium which was isolated from a terrestrial hydrothermal spring. M. humiferrea is able to use humic acid or 10-anthraquinone-2,6-disulfonate as an electron-shuttling compound for growth and Fe(III) reduction. The genome has a size of 2.629 Mb and contains 2,668 predicted protein-coding genes.

## GENOME ANNOUNCEMENT

Moorella spp. are known thermophilic acetogens and are of interest as sources of biocatalysts for biotechnological processes (1). Moorella humiferrea is a thermophilic bacterium with straight rod-shaped cells. Moreover, the cells are 0.3 to 0.5 µm in diameter and 2.0 to 5.0 µm in length, and M. humiferrea is motile via peritrichous flagella. M. humiferrea DSM 23265 was isolated from a terrestrial hot spring from the Grot geyser in Kamchatka, Russia (2). In the presence of 10-anthraquinone-2,6-disulfonate (AQDS) or humic acid, it is capable of growing and reducing Fe(III) (2).

The MasterPure complete DNA purification kit (Epicentre, Madison, WI, USA) was used to isolate the chromosomal DNA of M. humiferrea DSM 23265. The purified DNA was applied to produce Illumina paired-end sequencing libraries, as recommended by the manufacturer (Illumina, San Diego, CA, USA). Sequencing was performed with a MiSeq instrument and MiSeq reagent kit version 3 (Illumina). For quality control and quality filtering of the generated Illumina reads, FastQC version 0.11.5 (3) and Trimmomatic version 0.36 (4) were used, respectively. The recovered 1,335,382 high-quality paired-end reads were used for assembly. Genome assembly was performed with the SPAdes genome assembler software version 3.10.0 (5), yielding 63 contigs (>500 bp) and an average coverage of 103-fold. To validate the *de novo* assembly, Qualimap version 2.1 was employed (6). The draft genome was 2.629 Mb, with a GC content of 53.52%. For gene prediction and annotation, the software tool Prokka was employed (7). Gene prediction yielded 2,668 protein-coding genes, of which 2,077 genes were with functional annotations. Additionally, 3 rRNA genes, 49 tRNA genes, and 1 transfer-messenger RNA (tmRNA) were identified. Eleven putative protein-coding genes were related to clustered regularly interspaced short palindromic repeat (CRISPR)/CRISPR-associated (Cas) systems. Furthermore, the genome harbored 8 putative genes coding for multidrug resistances.

In the presence of AQDS, M. humiferrea is able to utilize lactate, malate, succinate, glycerol, and yeast extract as substrates (2). The oxidation of lactate is incomplete, with acetate as the sole metabolic product (2). Two putative genes for acetate kinases were present in the genome, but the *pta* gene coding for a phosphotransacetylase was not identified. Nevertheless, one *pduL* gene, which could be responsible for the transacetylase activity (8), was detected in close vicinity of one acetate kinase-encoding gene. In compliance with other Moorella genomes, the sequences encoding proteins of the Wood-Ljungdahl pathway of M. humiferrea DSM 23265 were organized as gene clusters. Remarkably, the carbonyl branch of this pathway corresponded to the gene cluster present in M. thermoacetica strains DSM 2955 (9) and DSM 521 (10), supporting the hypothesis that this cluster is highly conserved among closely related species (11). Several genes coding for oxidoreductases, hydrolases, and aminopeptidases probably involved in humic acid degradation (12) were detected in the genome.

### Accession number(s).

The whole-genome shotgun project has been deposited at DDBJ/ENA/GenBank under the accession number PVXM00000000. The version described here is version PVXM01000000.
